# Therapist and client perspectives on the alliance in the treatment of traumatized adolescents

**DOI:** 10.3402/ejpt.v6.27705

**Published:** 2015-08-31

**Authors:** Silje M. Ormhaug, Stephen R. Shirk, Tore Wentzel-Larsen

**Affiliations:** 1 Norwegian Centre for Violence and Traumatic Stress Studies (NKVTS), Oslo, Norway; 2 Department of Psychology, University of Oslo, Oslo, Norway; 3 Department of Psychology, University of Denver, Denver, CO, USA; 4 Centre for Child and Adolescent Mental Health, Eastern and Southern Norway (RBUP), Oslo, Norway

**Keywords:** Working alliance, youth and therapist perspectives, discrepant alliance ratings, posttraumatic stress disorder, exploratory factor analyses

## Abstract

**Objective:**

Client ratings of the therapeutic alliance are an important predictor of outcome in the treatment of traumatized adolescents and adults, but less is known about the therapists’ perspective. The aim of this study was to investigate how therapists’ ratings relate to the adolescents’ perspective, how individual therapist and adolescent ratings relate to change in symptoms and treatment satisfaction, and whether discrepant alliance perspectives impact treatment outcome.

**Method:**

The sample consisted of 156 youth (mean age 15.1, range 10–18), randomized to trauma-focused cognitive behavioral therapy or treatment as usual, and alliance ratings from 62 therapists. Alliance was measured midtreatment with the Therapeutic Alliance Scale for Children, and the factor structure of the two scales was analyzed with exploratory factor analyses. A change in posttraumatic symptoms was assessed with the Child PTSD Symptom Scale (CPSS) and the Clinicial-Administered PTSD Scale for Children and Adolescents (CAPS-CA).

**Results:**

Therapist and client perspectives on the alliance were significantly, but moderately, associated (intraclass correlations [ICC]=0.54, *p<*0.001). Both scales predicted adolescent treatment satisfaction but only the client scale was significantly related to change in symptoms. Factor analyses revealed differences in factor structure with therapist ratings organized around bond and task dimensions and adolescent ratings organized by item valence. Higher therapist ratings compared to adolescent ratings predicted higher residual PTS symptoms.

**Discussion:**

Although adolescent and therapist alliance ratings are moderately associated, results suggest that the ratings are differentially associated with outcomes. These findings, along with results indicating important differences in factor structure, imply that adolescent and therapist ratings are not interchangeable. Future studies should investigate how therapists can improve their judgments of adolescents’ perceptions of the alliance as an overestimation of the quality of the relationship seems to be negatively related to outcome.

The therapeutic alliance is an important predictor of outcome in psychotherapy with children and adolescents (McLeod, 2011; Shirk, Karver, & Brown, 2011). In particular, the development of a strong, therapeutic alliance has been viewed as essential for the successful treatment of traumatized adolescents. This is, in part, because the experience of interpersonal trauma can alter core beliefs about others’ trustworthiness, thereby making treatment collaboration particularly challenging (Cloitre, Cohen, & Scarvalone, 2002; Cohen, Mannarino, Kliethermes, & Murray, 2012; Shirk & Eltz, 1998). In addition, treatment methods, especially the use of exposure techniques, may be perceived as demanding, and strain the working relationship between adolescent and therapist. Several studies of traumatized adults have found that a strong alliance is related to significantly lower symptom-level post-treatment (Cloitre, Koenen, Cohen, & Han, 2002; Cloitre, Stovall-McClough, Miranda, & Chemtob, 2004; Cronin, Brand, & Mattanah, 2014; McLaughlin, Keller, Feeny, Youngstrom, & Zoellner, 2014), findings that are in line with at least two studies with traumatized adolescents (Eltz, Shirk, & Sarlin, 1995; Ormhaug, Jensen, Wentzel-Larsen, & Shirk, 2014).

The majority of these studies focused on the client perspective of the alliance. Only two included therapist alliance ratings (Cronin et al., 2014; Eltz et al., 1995). Consequently, less is known about therapists’ perspectives on the alliance, and how this corresponds to clients’ views in trauma treatments. Clinically, therapists’ perspectives are important because the therapists are responsible for managing the therapeutic process, including handling potential alliance ruptures (Safran, Muran, & Eubanks-Carter, 2011). Furthermore, the therapists’ evaluations of the alliance are likely to influence in-session decision-making about specific interventions. For example, if a therapist perceives a fragile alliance, he or she may be reluctant to press the client to engage in challenging treatment tasks such as exposure. In fact, there is some evidence that concerns about alliance rupture is one of the primary reasons therapists avoid exposure-based interventions (Kendall et al., 2009). As studies indicate that exposure tasks are important to reduce posttraumatic stress disorder (PTSD) symptoms (Deblinger, Mannarino, Cohen, Runyon, & Steer, 2011; Ehlers et al., 2010), omitting this component may potentially reduce treatment effectiveness. On the other hand, if therapists overestimate alliance strength relative to their adolescent clients, they may fail to engage in supportive strategies to maintain treatment collaboration. In fact, failure to recognize the adolescent's perspectives on the alliance, as reflected in discrepant alliance ratings, could indicate a lack of therapist attunement to the adolescent's experience and predict poorer outcomes. In the adult therapy literature, it has been found that low levels of client–therapist agreement on the alliance are related to lower session smoothness and less symptom change (Marmarosh & Kivlighan, 2012). Also, lack of therapist attunement to, and reparation of, alliance ruptures have been related to poorer outcomes (McLaughlin et al., 2014; Safran et al., 2011). There are, however, to our knowledge, no studies that have investigated the potential impact of discrepant therapist and adolescent perspectives on process and outcomes.

Several studies have shown that the level of youth and therapist agreement on the alliance is small to moderate (Accurso & Garland, 2015; Creed & Kendall, 2005; Eltz et al., 1995; Hawley & Garland, 2008; Kendall et al., 2009). This is consistent with the findings from the adult field, where a meta-analysis showed that the average correlation between therapist and client perspectives was *r=*0.36 (Tryon, Blackwell, & Hammel, 2007). Although this suggests some level of convergence between the two perspectives, it also points to important differences between the raters’ views of the alliance. In the adult treatment literature, there is evidence for differences in the underlying factor structure of therapist and client alliance ratings (Bachelor, 2013). Although less studied, research on youth alliance has indicated that child and adolescent ratings of the alliance may differ from therapist ratings and not conform to the conceptual model advanced by Bordin (1979) with distinct factors related to bond, task, and goals. Instead, DiGiuseppe, Linscott, and Jilton (1996) found adolescent ratings to be unidimensional. Interestingly, therapist ratings reflected the multidimensional structure proposed by Bordin (1979). In another study of the Therapeutic Alliance Scale for Children—revised (TASC-r; Shirk & Karver, 2010; Shirk & Saiz, 1992), it was found that child and adolescent ratings were organized by item valence rather than by the hypothesized dimensions of bond and task (Accurso, Hawley, & Garland, 2013). Therefore, discrepancies in youth and therapist ratings of the alliance might represent more than differences in vantage point; they could reflect differences in conceptualization of alliance.

The primary aim of this study was to examine therapist evaluations of alliance in the treatment of traumatized adolescents. Specifically, the degree of correspondence between therapist and client ratings of alliance was examined, and the degree to which each perspective predicted treatment outcome was assessed. Because shared source variance may represent confound in alliance—outcome studies, both adolescent and therapist ratings of alliance were examined as predictors of outcomes assessed from adolescent and clinician perspectives. In addition to symptom change, adolescents’ satisfaction with treatment was also evaluated. Given prior results indicating only moderate associations between client and therapist ratings, potential factors underlying differences in therapist and youth ratings were explored. Specifically, the factor structure of adolescent and therapist ratings were evaluated to examine whether the treatment participants perceive the alliance as a similar construct. Finally, we examined whether discrepant views of alliance were associated with treatment outcomes. It was hypothesized that greater divergence between therapist and adolescent ratings of alliance signals poor therapist attunement to the adolescent's experience and represents a marker of negative therapeutic process. Although absolute discrepancy could indicate attunement lapses, we expected that lower adolescent than therapist ratings would be at greater risk for poorer outcomes.

## Methods

### Procedure

Data were derived from a randomized effectiveness study comparing trauma-focused cognitive behavioral therapy (TF-CBT; Cohen, Mannarino, & Deblinger, 2006) to treatment as usual (TAU) for traumatized youth (Jensen et al., 2014). All participants were referred and received treatment at one of eight community outpatient clinics in Norway. Inclusion criteria were clinically significant levels of posttraumatic stress symptoms (PTSS) (≥15 on the Child Posttraumatic Symptom Scale [CPSS]; Foa, Johnson, Feeny, & Treadwell, 2001). Exclusion criteria were psychotic disorders, suicidal behavior, or need of an interpreter. All procedures were reviewed and approved by the Regional Committee for Medical and Health Research, and written consent was obtained from both the caretaker and the adolescent. Because the alliance has been conceptualized as a measure of how well the client and therapist work together (Horvath, Del Re, Flückiger, & Symonds, 2011), the alliance was assessed after session 6. This was to ensure that the adolescents had established an opinion of their working collaboration with the therapist. Post-treatment PTS symptom-level and adolescents’ satisfaction with the treatment was assessed after 15 sessions. Licensed psychologists from the study group conducted all assessments, and adolescents were informed that the therapists would not be able to see their alliance ratings. For a more detailed description about the study and the treatment conditions, see Jensen et al. (2014).

### Participants

#### Youth sample

A total of 156 adolescents (mean age 15.1, SD: 2.2, range: 10–18) participated in this study, of which 130 completed the session 6 alliance rating. Half of these adolescents (*n=*65) were randomized to the TF-CBT condition, and the other half to TAU. The majority was female (80.8%) and had at least one Norwegian born parent (83.9%). Adolescents reported on average 3.5 different types of traumatizing events (SD: 1.6, range 1–8). The most commonly reported events were: sudden death of a caregiver or close person (58.8%), physical assault outside the family (58.0%), witnessing violence and physical abuse within the family (43.5%), sexual abuse outside the family (28.2%), and witnessing violence outside the family (26.0%). The 26 adolescents that did not complete the alliance ratings were not significantly different from the 130 completers in terms of age, sex, number of traumatic events, or pretreatment PTS symptoms. For more detailed information about the adolescent sample, see Ormhaug et al. (2014).

#### Therapist sample

In total, 71 therapists volunteered to participate in the study. Of these, 62 rated their alliance with 126 adolescents. This sample consisted of 120 therapy dyads, and 6 therapists with no corresponding adolescent ratings. On average, each therapist rated the alliance with two youth (SD: 1.2, range 1–6). The therapist group was predominantly female (87.3%) and consisted of 39 (62.9%) psychologists, 10 (16.1%) educational therapists, 9 (14.5%) social workers, and 4 (6.4%) psychiatrists. Therapists reported on average 12.1 years of postgraduate working experience (SD: 9.7, median=9.0, range 1–40). Adolescents whose therapists did not report an alliance rating (*n=*30) were significantly older (mean age 15.8 vs. 14.9, *t*[154]=2.1, *p=*0.039) and reported significantly higher pretreatment CPSS scores (mean score 29.8 vs. 26.4, *t*[154]=2.2, *p=*0.028) compared to adolescents with therapist-rated alliance scores.

### Alliance measures

#### Adolescent-rated alliance

TASC-r (Shirk & Karver, 2010; Shirk & Saiz 1992) is a self-reported scale that includes 12 items. These are worded as statements regarding the youths’ feelings towards the therapist (e.g., “I like my therapist”) and their self-perceived involvement in therapeutic tasks (e.g., “I work with my therapist to solve problems in my life”). Six of the items are developed to cover the bond-aspect of the alliance (items 1, 3, 5, 6, 8, and 10) and six items to relate to the tasks of therapy (items 2, 4, 7, 9, 11, and 12). All items are answered on a 4-point Likert scale (Not at all to Very much). The scale has demonstrated good reliability and validity in previous studies (Accurso & Garland, 2015; Creed & Kendall, 2005; Kendall et al., 2009). In this study, the TASC-r was translated and back translated, and the first author of the TASC-r approved the Norwegian version. Internal reliability was good in this sample (*α*=0.91, Raykov's reliability index [Raykov, 2009]: 0.92 [95% CI: 0.89–0.94]).

#### Therapist-rated alliance

Therapists’ perspectives on the alliance were evaluated with the therapist version of the TASC-r. In this scale, items are phrased so that therapists rate their evaluation of the clients’ experience of the alliance (e.g., “The child expresses positive emotions towards you, the therapist”; “The child finds it hard to work with you on solving problems in his/her life”). The therapist scale has demonstrated good reliability in a previous study (Accurso & Garland, 2015) and had a good internal reliability in this sample (*α*=0.91, Raykov's reliability index: 0.91 [95% CI: 0.88–0.95]).

### Outcome measures

#### Self-reported PTSS

Adolescents’ PTSS were first measured with the Child PTSD Symptom Scale (CPSS; Foa et al., 2001). This scale measures the 17 symptoms of PTSD defined in the DSM-IV. Participants report the frequency of symptoms during the last 2 weeks, rated on a 4-point scale ranging from Never or once to Almost every day. The scale was translated and back-translated, and the developers of the scale approved the Norwegian version. Satisfactory internal consistency values were found for each of the three factors (Re-experience: *α*=0.84, Avoidance: *α*=0.80, Hyperarousal: *α*=0.76, Raykov's reliability indexes: total scale: 0.92 [95% CI: 0.90–0.94], Re-experience: 0.89 [95% CI: 0.84–0.92], Avoidance: 0.83 [95% CI: 0.78–0.87], Hypervigilance: 0.77 [95% CI: 0.71–0.84]).

##### Clinician-rated PTSS

The clinician-administered PTSD scale for children and adolescents (CAPS-CA; Nader et al., 2004) measures the frequency and intensity of the 17 DSM-IV defined symptoms of PTSD. Items are scored on 5-point frequency scales (e.g., from 0 [None of the time] to 4 [Most of the time]) and 5-point intensity rating scales (e.g., from 0 [Not a problem] to 4 [A big problem, I have to stop what I am doing]), assessing the past month. Items are scored based on both the youths’ answers and clinical judgment during the interview. The interview was translated and back translated, and the first author of the CAPS-CA approved the Norwegian version. The scale showed satisfactory internal consistency (total scale *α*=0.90, Re-experiencing: *α*=0.87, Avoidance: *α*=0.77, Hyperarousal: *α*=0.79). Inter-rater reliability was excellent (intraclass correlations [ICC]=0.99; 95% CI: 0.95–1.00), and κ value on diagnostic status was 0.80.

#### Adolescents’ treatment satisfaction

To assess adolescents’ satisfaction with the therapy, a three-item self-report measure was developed. Items were “I liked going to the clinic,” “Going to the clinic helped me with my problems,” and “If I were ever having problems again, I would want to come back to this clinic.” All items were rated on a 4-point scale form ranging from 1 (Not at all) to 4 (All of the time), and the scale was administered at the post-treatment assessment. Internal consistency of the scale was good (α: 0.85; Raykov's reliability index: 0.86 [95% CI: 0.81–0.91]).

### Data analyses

Level of agreement between adolescent and therapist ratings of the alliance was evaluated in terms of an effect size (ES: mean difference/pooled SD and ICC). Relationships between alliance scores and outcome were analyzed with linear regressions. Pretreatment symptom levels were included in the models predicting post-treatment symptom scores, and change scores (level of symptom change from pre to post) were included in the youth satisfaction model. Differences in correlations of youth and therapist perspectives were tested using bootstrap *BC*_*a*_ intervals (10,000 bootstrap replications). Factor structure of the two scales was investigated with exploratory factor analyses. Geomin factor loadings were used (Muthén & Muthén, 1998–2012), and extractions were specified to one or two factors. Because the TASC-r items are rated on a 4-point scale, items were entered as categorical (Rhemtulla, Brosseau-Liard, & Savalei, 2012). To determine model fit, both likelihood-ratio chi square and descriptive fit indices were utilized. The descriptive fit indices included the comparative fit index (CFI), the Tucker–Lewis fit index (TLI), the root-mean-square error of approximation (RMSEA), and the standardized root-mean-square residual (SRMR). These fit indices have most commonly been studied as indicators of structural equation modeling and confirmatory factor analyses (see, e.g., Schreiber, Stage, King, Nora, & Barlow, 2006) and there are currently no established cut-off values for the use of the fit indices in EFA (Barendse, Oort, & Timmerman, 2015). In our study, we followed Accurso et al. (2013) where models that fit very well (or adequately) were indicated by CFIs and TLIs ≥0.95 (0.90–0.94), RMSEAs <0.05 (to 0.08), and SRMR <0.05 (to 0.08). A model was determined to be well-fitting if three of the four descriptive indices indicated good fit. Because of the nested nature of the data the within subjects ICC were calculated to see whether the clinic and/or therapist level had to be taken into account in the EFA and regression analyses. It was assumed that multilevel models could be difficult or impossible to estimate with small ICCs and would not be beneficial with ICC levels lower than 0.05 (Dyer, Hanges, & Hall, 2005). Finally, to investigate whether discrepant views of the alliance were associated with treatment outcome, a difference score (therapist ratings minus youth ratings) was entered as a predictor in the regression models.

Preliminary analyses and analyses of cross-informant agreement were conducted using PASW Statistics 22.0 (IBM, 2014), and exploratory factor analyses were run in Mplus 7.0 (Muthén & Muthén, 1998–2012). The remaining analyses were run in R (The R Foundation for Statistical Computation, Vienna, Austria), with multiple imputations calculated using the R package mice (Van Buuren, 2012), and bootstrapping with the R package boot.

#### Missing data

Of the 156 recruited youth, 130 completed the midtreatment alliance ratings. As the missing data were not assumed to be completely at random, discarding data using listwise deletion could increase the risk of a biased result (Schafer & Graham, 2002). To account for missing values in covariates, regression analyses were repeated using multiple imputations (200 completed data sets, see R script, Supplementary file). Because analyses with imputed data yielded similar results to the complete-case analyses, reported results will be based on imputed material.^1^

## Results

### Preliminary analyses

There were no significant differences in neither adolescent nor therapist TASC-r scores across gender, minority status (in absolute value: *t’*s ≤1.6; *p’*s ≥0.105), or therapist education (*F’*s ≤1.6, *p’*s ≥0.174). Correlations between TASC-r scores and pretreatment PTSS scores were weak and not significant (in absolute value: *r’*s ≤0.15; *p’*s ≥0.144). There were no significant differences in alliance scores between the two treatment conditions (Table 1).

**Table 1:** Adolescent and therapist ratings of the alliance across treatment conditions

Scale	Group	*n*	Mean score	SD^a^	Df	*t*	*p*
Adolescent-rated TASC-r	TF-CBT TAU	65 65	38.1 38.0	7.6 8.0	128	0.5	0.964
Therapist-rated TASC-r	TF-CBT TAU	64 62	35.9 34.9	5.4 7.0	124	0.9	0.364
Therapist—adolescent discrepancy	TF-CBT TAU	61 59	−1.9 −2.9	8.6 7.0	118	0.7	0.490

TASC-r, therapeutic alliance scale for children revised.

a Test of homogeneity of variance: all Levene *p*≥0.05.

### Cross-informant agreement and mean alliance scores

**Fig. 1:**
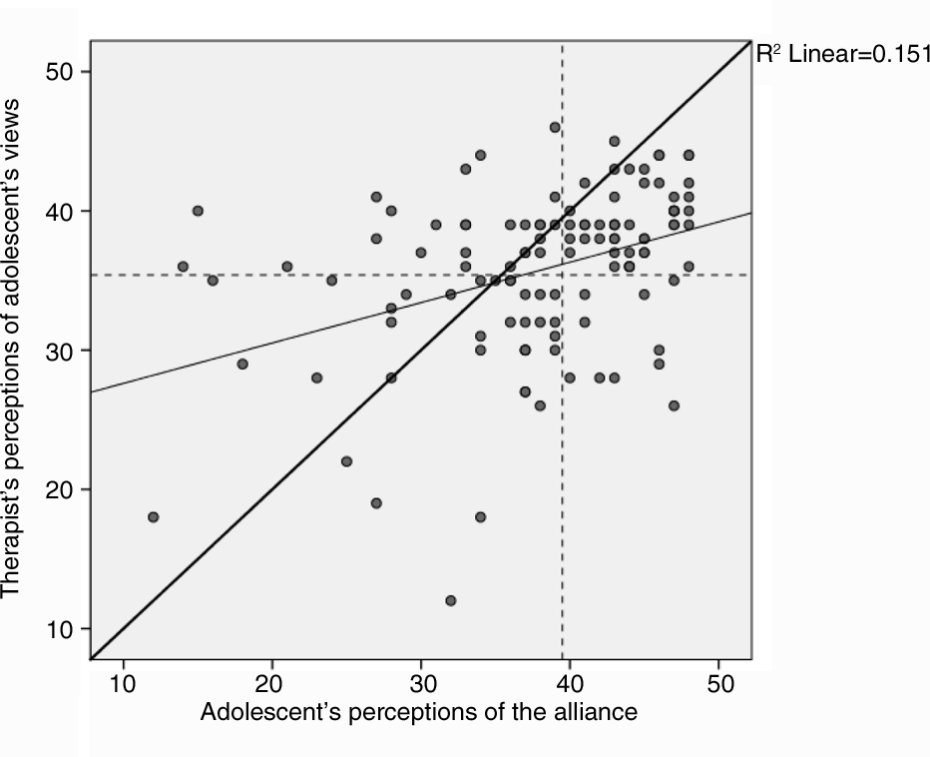
Divergence of therapists’ and adolescents’ alliance ratings. Diagonal thick line=no divergence between therapist and adolescent ratings; diagonal thin line=regression line; dashed horizontal line=mean therapist ratings; and dashed vertical line=mean adolescent rating.

The level of client–therapist agreement was moderate (ICC=0.54, *p<*0.001). On average, adolescents reported higher alliance scores compared to therapists (*M*=38.2, SD: 7.9 vs. *M=*35.8, SD: 5.9; *t*[119]=3.4, *p*=0.001). This difference corresponds to an ES of *d=*0.37. Absolute mean discrepancy was 2.4 (SD: 7.9). As shown in Fig. 1, there was a tendency that therapists underestimated adolescent's high alliance scores (indicated by points to the right of the adolescent mean and below the diagonal line) and overestimated adolescent's low alliance scores (points to the left of the adolescent mean and above the diagonal line; Fig. 1).

### Alliance—outcome process relations

Investigations of the ICCs showed that the variance by clinic was ignorable (<0.05), but the variance between-therapists ICCs ranged from 0.10 to 0.24, with an average ICC of 0.17. In an attempt to take this variance into account, analyses were first conducted with linear mixed models with adolescents nested within therapist. Results showed, however, that the models came out unstable and subsequent analyses were performed with single-level analyses. Regressions showed that adolescent ratings of alliance significantly predicted both self-reported PTSS post-treatment and was marginally associated with clinician-rated PTSS (*p=*0.095) after controlling for pretreatment symptoms (Table 2). In addition, adolescent ratings predicted treatment satisfaction. Therapist ratings significantly predicted adolescents’ treatment satisfaction but were not significantly related to neither adolescent nor clinical reports of post-treatment symptoms. The relationships between adolescents’ and therapists’ alliance ratings and changes in PTSS were small (0<*r’*s ≤0.15), but the relationships to adolescents’ treatment satisfaction were moderate to large (0<*r*s ≥0.43; Table 3). Tests of magnitude of effects showed that the differences in adolescents’ versus therapists’ perspectives were not statistically significant neither on adolescent-rated symptom change (*M* difference=0.17, 95% CI: −0.00 to 0.35) nor on the clinician-rated symptom change (*M* difference=0.17, 95% CI: −0.03 to 0.38).

**Table 2:** Linear regressions with adolescent and therapist alliance ratings and outcome

	Est.	95% CI	*p*	Stand. est.^a^
Adolescent-rated PTSS posttreatment				
Pretreatment CPSS	0.48	0.23–0.72	<0. 001	0.32
Adolescent-rated alliance	−0.33	−0.61−− 0.04	0.024	−0.22
Therapist-rated alliance	0.29	−0.09–0.67	0.138	0.13
Clinical-rated PTSS post-treatment				
Pretreatment CAPS-CA	0.60	0.40–0.81	<0.001	0.46
Adolescent-rated alliance	−0.58	−1.26–0.10	0.092	−0.15
Therapist-rated alliance	0.75	−0.22–1.72	0.129	0.14
Adolescents’ treatment satisfaction				
Change in CPSS scores pre-post	0.06	0.03–0.10	<0.001	0.17
Adolescent-rated alliance	0.15	0.09–0.21	<0.001	0.29
Therapist-rated alliance	0.14	0.06–0.22	<0.001	0.60

CPSS, Child PTS Symptom scale; CAPS-CA, clinical diagnostic interview of adolescents’ PTSS. Table presented with multiply imputed data.

a Standardized estimates are based on regression coefficients from imputed data and standard deviations from complete cases.

**Table 3:** Correlations between alliance scales and outcome measures

	1	2	3	4	5
1. Adolescent-rated alliance					
2. Therapist-rated alliance	0.39***				
3. Change in CPSS pre-post	0.15	−0.02			
4. Change in CAPS-CA pre-post	0.13	−0.04	0.68***		
5. Child satisfaction	0.56***	0.43***	0.32***	0.24**	

CPSS, Child PTS Symptom scale; CAPS-CA, clinical diagnostic interview of adolescents’ PTSS.

*** *p*<0.001

** *p<*0.010

* *p<*0.050.

### Factor structure of adolescent and therapist alliance

#### Adolescent scale

Because of nesting, an attempt was made to take the therapist variance into account and analyses were first conducted with multilevel EFA. However, the models were unstable and the factor analyses were performed using single-level EFA. For the adolescent scale, a two-factor solution yielded the best result (*χ* ^2^ one-factor: 208.3 [54 df], *p<*0.001; two-factor: 81.6 [43 df], *p<*0.001; comparison between models: *χ* ^2^: 94.4, *p<*0.001). Inspection of the descriptive fit indices showed that this model was acceptable (CFI=0.97; TLI=0.98; SRMR=0.05; RMSEA=0.08). Seven of the items loaded on the first factor (items 1, 2, 5, 6, 7, 8, and 11) and the remaining five items (3, 4, 9, 10, and 12) loaded on the second factor. As five of the seven items in the first factor were negatively worded, this was named Adolescent Negative, and the second factor was named Adolescent Positive (Table 4).

**Table 4:** Factor loadings for exploratory factor analyses with geomin rotation for adolescent and therapist TASC-r scales

	Adolescent scale		Therapist scale	
		
Item	Factor 1	Factor 2	Factor 1	Factor 2
Item 1	0.64*	0.29*	0.99*	−0.08
Item 2r	0.49*	0.29*	0.13	0.56*
Item 3	0.27*	0.65*	0.78*	0.14
Item 4	0.00	0.88*	0.00	0.95*
Item 5r	0.93*	−0.16	0.52*	0.31
Item 6	0.77*	0.10	0.69*	0.06
Item 7r	0.62*	0.05	−0.05	0.74*
Item 8r	0.85*	0.00	0.70*	0.16
Item 9	−0.13	0.92*	−0.19	1.00*
Item 10	0.35*	0.64*	0.91*	−0.00
Item 11r	0.76*	0.05	0.07	0.77*
Item 12	0.19	0.74*	0.02	0.93*

Geomin rotated loadings, items entered as categorical.

* *p <* 0.050. Factor loadings ≥0.40 are shown in bold. *r* specifies that the item is negatively worded and the scoring of the item is reversed.

#### Therapist scale

Also for the therapist scale, a two-factor solution showed the best fit (*χ* ^2^ one-factor: 343.8 [54 df], *p*<0.001; two-factor: 152.6 [43 df], *p*<0.001; comparisons between models: *χ* ^2^: 140.6 [11 df], *p<*0.001). The descriptive fit indices for the two-factor model were adequate (CFI=0.97; TLI=0.95; SRMR=0.07; RMSEA=0.14). Each factor comprised six items, with items in the first factor (1, 3, 5, 6, 8, 10) corresponding to the bond dimension (Therapist Bond) and items in the second factor (2, 4, 7, 9, 11, 12) corresponding to the task dimension (Therapist Task) (Table 4).

### Therapist-client discrepancy and outcomes

The final analyses showed that higher therapist–client discrepancies in alliance ratings significantly predicted higher adolescent- and clinician-rated post-treatment symptom scores. Discrepancy was marginally related to lower adolescent-rated satisfaction with services (Table 5).

**Table 5:** Linear regressions for therapist—adolescent discrepancy and outcome

	Est.	95% CI	*p*	Std est.^a^
Adolescent-rated PTSS post-treatment (*n*=120)				
Pretreatment CPSS	0.47	0.23–0.71	<0.001	0.31
Therapist—adolescent discrepancy	0.32	0.05–0.59	0.020	0.22
Clinician-rated PTSS post-treatment (*n=*110)				
Pretreatment CAPS-CA	0.60	0.40–0.81	<0.001	0.45
Therapist—adolescent discrepancy	0.63	−0.00–1.26	0.050	0.17
Adolescents’ treatment satisfaction (*n=*112)				
Change in CPSS scores pre—post	0.06	0.02–0.11	0.004	0.28
Therapist—adolescent discrepancy	−0.06	−0.13–0.00	0.061	−0.17

CPSS, Child PTS Symptom scale; CAPS-CA, clinical diagnostic interview of adolescents’ PTSS. Table presented with multiply imputed data.

a Standardized estimates are based on regression coefficients from imputed data and standard deviations from complete cases.

## Discussion

Although a strong therapeutic alliance has been linked to better outcomes in the treatment of traumatized adolescents and adults, prior research has mainly focused on the client's view of alliance and its relation to outcome. In this study, the relationship between client and therapist alliance perspectives, their association with outcomes, and the impact of alliance discrepancy were evaluated in a sample of traumatized adolescents undergoing treatment. Consistent with prior studies on the TASC-r, it was found that the two alliance perspectives were moderately associated (Accurso & Garland, 2015; Creed & Kendall, 2005; Fjermestad et al., 2012; Kendall et al., 2009). Also, similar to studies on adults (Hartmann, Joos, Orlinsky, & Zeek, 2015; Tryon et al., 2007) therapists rated the alliance less positively than clients. Given that the TASC-r requires therapists to view the alliance from their client's perspective, this pattern indicates that therapists underestimate alliance strength relative to their adolescent clients. It has been suggested that discrepancies in alliance ratings may be related to differences in clients’ and therapists’ implicit views of the alliance construct (Bachelor, 2013). Current results showed that the factor structure of the TASC-r differed across therapist and client perspectives. Findings indicated that therapist ratings clustered based on item content, consistent with dimensions hypothesized by Bordin (1979). However, adolescent ratings of alliance did not separate into bond and task dimensions but were instead structured along item valence, that is, whether they are positively or negatively worded. This pattern for adolescent ratings is consistent with the recent results obtained by Accurso et al. (2013) and suggests that the alliance is largely an affective construct for children and adolescents. It may be that therapists, who often have a broader reference group of patients, judge their clients’ alliance based on their collaborative involvement in the therapeutic tasks. Clients, on the other hand, are likely to compare therapists’ attentive and empathic attitudes to responses they usually receive from friends and family members (Hartmann et al., 2015), and this may have a positive influence on their alliance ratings.

In this study, client and therapist alliance ratings showed different associations with change in posttraumatic stress symptoms. Consistent with prior research (Cloitre, Koenen, et al., 2002; Cloitre et al., 2004; Cronin et al., 2014; McLaughlin et al., 2014), adolescent alliance ratings were significantly associated with self-reported change in PTSS and were marginally related to clinician reports of symptom change. In contrast, therapist ratings were not significantly related to either measure of symptom change, but comparisons of magnitude in correlations showed that client and therapist associations with adolescent-rated symptom change were not significantly different. However, like adolescent ratings of alliance, therapist ratings did predict client treatment satisfaction. This pattern of findings suggests that a positive relationship between adolescent and therapist is an important component of satisfaction with therapy process, and corresponds to prior research that has found that therapist perspectives are related to treatment progress in therapy with traumatized clients (Cronin et al., 2014; Eltz et al., 1995). Although it has been claimed that treatment satisfaction is not a good indicator of clinical change (Garland, Aarons, Hawley, & Hough, 2003), in this study there was a significant association between treatment satisfaction and symptom change. In fact, when considered together, both symptom change and alliance predicted client satisfaction.

Results also provided some evidence that the level of discrepancy between therapist and client views of alliance was associated with outcome and satisfaction. Adolescents whose therapists rated the alliance as relatively more positive than the adolescents showed less symptom reduction compared to dyads where alliance ratings were similar or more positively rated by the adolescent. This pattern is intriguing in that more positive therapist ratings of alliance predict poorer outcomes when considered in relation to client ratings. It is possible that therapists who overestimate alliance strength relative to their adolescent clients are less attuned to their client's experiences in therapy. This could lead them to overlook alliance problems that could impact treatment progress. Alternatively, adolescents who view the alliance more negatively than their therapists may be less open and more difficult to read by their therapists. Either way, these results suggest that future research should include both client and therapist perspectives on the alliance, not simply because they are not interchangeable, but because their level of convergence or discrepancy within dyads appears to predict outcome.

Although this study has a number of strengths including a relatively large referred adolescent sample treated by community clinicians, and the inclusion of different perspectives on both alliance and outcome, a number of limitations must be considered. The first concerns the non-independence of the alliance ratings as youth were nested within therapists. Investigations of the ICC levels indicated that multilevel analyses would be warranted; however, efforts to take this nesting into account produced unstable estimates and were not computed. This could be due to the distribution of youth–therapist pairs in this sample as 43.5% of the therapists rated their alliance with one youth only, and only 30.7% rated their alliance with three or more youth. Nevertheless, the use of single-level analyses means that a certain amount of doubt concerning our findings cannot be ruled out. Furthermore, only alliance ratings from one time point were included in this study. As a fluctuation in the alliance over time has been found to be significantly related to the outcome of PTSD treatment (McLaughlin et al., 2014), the inclusion of more assessment points would also have strengthened the results of our study. In this study, the alliance was assessed at session 6. This is a time point where most participants in the TF-CBT condition had started their work on the trauma narrative (Cohen et al., 2006), and it cannot be ruled out that this potentially demanding task may have influenced the adolescents’ alliance ratings. However, analyses showed that in TAU, the treatment condition that did not include specified work on a trauma narrative (Ormhaug et al., 2014), the alliance ratings were comparable to the TF-CBT participants’ ratings. This finding could indicate that the trauma narrative did not have extensive influence on the alliance ratings. Also, the rates of missing data for several predictor variables may have biased our results. Although analyses with multiple imputations showed similar results as complete case analyses, indicating that the impact of missing data was not substantial, results would have been stronger if our data were more complete. The final limitation refers to the composition of the sample. This study had a predominantly female sample, and although there was some ethnic diversity in the sample, most of the participants were of Norwegian descent. Limited diversity could affect the ability to generalize from our results.

Findings showing important differences between therapist and adolescent ratings of alliance carry a number of implications. From a research perspective, therapist and adolescent perspectives are not interchangeable. The finding that therapist and youth reports are potentially based on two different conceptual models of the alliance has implications for how youth and therapist ratings are interpreted. Adolescents appear to view the alliance in more general affective terms, whereas therapists distinguish therapy work from relational bond. Perhaps, it is not surprising, then, that adolescent and therapist alliance ratings are only moderately associated and have somewhat different associations with change in posttraumatic symptoms. Although it is tempting to recommend that only the client perspective be used as a predictor of outcome, the degree of discrepancy between therapist and adolescent ratings also proved to be related to symptom change. Future research should examine the impact of discrepancy on treatment process to uncover possible mediating variables, for example, the impact on treatment fidelity. From a clinical perspective, the prevalence of therapist–adolescent discrepancy suggests that it might be useful for therapists to receive direct feedback on alliance over the course of treatment. Like feedback on symptom change, alliance feedback could help therapists know when treatment is “off track.” Finally, and of specific relevance to the treatment of traumatized adolescents, alliance formation and maintenance appear to be a critical component of successful therapy. Future research should address therapist strategies that promote and maintain the therapeutic alliance with traumatized youth and the impact of alliance level on implementation of core treatment components such as exposure and cognitive restructuring.

## Supplementary Material

Therapist and client perspectives on the alliance in the treatment of traumatized adolescents

Therapist and client perspectives on the alliance in the treatment of traumatized adolescents

## Footnotes

1 Results from complete case analyses can be made available upon request from the first author.
