# Supplementary material for: Therapist and client perspectives on the alliance in the treatment of traumatized adolescents
Source: Eur J Psychotraumatol. 2015 Aug 31;6:10.3402/ejpt.v6.27705. doi: 10.3402/ejpt.v6.27705 (PMC4557093; doi:10.3402/ejpt.v6.27705)
Supplement: Therapist and client perspectives on the alliance in the treatment of traumatized adolescents [file EJPT-6-27705-s001.pdf]

Mplus syntax for exploratory factor analyses (EFA) of the Therapeutic Alliance Scale-R (TASC-R) reported in the study:
"Therapist and Client Perspectives on the Alliance in the Treatment of Traumatized Adolescents"

```
TITLE: EFA of the Youth TASC-R, categorical variables

VARIABLE: NAMES ARE Id Gr Ch_1 Ch_2r Ch_3 Ch_4 Ch_5r Ch_6 Ch_7r
        Ch_8r  Ch_9   Ch_10  Ch_11r Ch_12;
   MISSING IS Ch_1   Ch_2r  Ch_3 Ch_4 Ch_5r Ch_6 Ch_7r
        Ch_8r  Ch_9   Ch_10  Ch_11r Ch_12 (999);
   USEV Ch_1  Ch_2r  Ch_3 Ch_4 Ch_5r Ch_6 Ch_7r
        Ch_8r  Ch_9   Ch_10  Ch_11r Ch_12;
   CATEGORICAL ARE Ch_1  Ch_2r  Ch_3 Ch_4 Ch_5r Ch_6 Ch_7r
        Ch_8r  Ch_9   Ch_10  Ch_11r Ch_12;
ANALYSIS: TYPE = EFA 1 2;
```

```
TITLE: EFA of the therapist TASC-R, categorical variables

VARIABLE: NAMES ARE Id Gr Ther_1 Ther_2r Ther_3 Ther_4
        Ther_5r Ther_6 Ther_7r Ther_8r Ther_9   Ther_10  Ther_11r  Ther_12;
 MISSING IS    Ther_1 Ther_2r Ther_3 Ther_4 Ther_5r Ther_6
        Ther_7r Ther_8r Ther_9 Ther_10 Ther_11r Ther_12 (999);
   USEV Ther_1 Ther_2r Ther_3 Ther_4 Ther_5r Ther_6
        Ther_7r Ther_8r Ther_9 Ther_10 Ther_11r Ther_12;
   CATEGORICAL ARE Ther_1 Ther_2r Ther_3 Ther_4 Ther_5r Ther_6
        Ther_7r Ther_8r Ther_9 Ther_10 Ther_11r Ther_12;
  ANALYSIS: TYPE = EFA 1 2;
```
