# Supplementary material for: Therapist and client perspectives on the alliance in the treatment of traumatized adolescents
Source: Eur J Psychotraumatol. 2015 Aug 31;6:10.3402/ejpt.v6.27705. doi: 10.3402/ejpt.v6.27705 (PMC4557093; doi:10.3402/ejpt.v6.27705)
Supplement: Therapist and client perspectives on the alliance in the treatment of traumatized adolescents [file EJPT-6-27705-s002.pdf]

R script for multiple imputations, regression analyses, and bootstrapping in the study "Therapist and Client Perspectives on the Alliance in the Treatment of Traumatized Adolescents"

#Preparing multiple imputation, including cases with complete outcome assessments

#Outcome: CPSS T3

```
ODScp3 <- ODS[is.na(ODS$CPSS_t3)==0,]

ODScp3.200 <- ODScp3[, c('Gender', 'Age', 'Group', 'CPSS_t1', 'CPSS_t2','CPSS_t3',
               'CAPS_T1', 'CAPS_T3',
               'YouthTASC_t2', 'TherTASC_t2',
                 'TherMinYouth',
               'Satisf.Child')]

ImpODScp3.200 <- mice(ODScp3.200, m = 200)
```

Outcome: CAPS-CA T3

```
ODScaps <- ODS[is.na(ODS$CAPS_T3)==0,]

ODScaps.200 <- ODScaps[, c('Gender', 'Age', 'CPSS_t1', 'CPSS_t2','CPSS_t3',
               'CAPS_T1', 'CAPS_T3',
               'YouthTASC_t2', 'TherTASC_t2',
               'TherMinYouth',
               'Satisf.Child')]

ImpODScaps.200 <- mice(ODScaps.200, m = 200)
```

Outcome: Child satisfaction

```
ODSchs <- ODS[is.na(ODS$Satisf.Child)==0,]

ODSchs.200 <- ODSchs[, c('Gender', 'Age', 'Group', 'CPSS_t1', 'CPSS_t2','CPSS_t3',
               'CAPS_T1', 'CAPS_T3',
               'YouthTASC_t2', 'TherTASC_t2', 'YouthChange', 'TherChange',
               'TherMinYouth',
               'Satisf.Child',
                'Ch_CPSSt1t3')]

ImpODSchs.200 <- mice(ODSchs.200, m = 200)
```

##Regression analyses

Model 1: DV= CPSS t3, IV= Youth alliance t2, Therapist alliance t2, CPSS t1

```
lm.cps3.1yt <- lm.mids(CPSS_t3~ CPSS_t1+YouthTASC_t2 + TherTASC_t2,
              data=ImpODScp3.200)
Pool.lm.cps3.1yt <- pool(lm.cps3.1yt)
summary(Pool.lm.cps3.1yt)
```

Calculating standardized coefficients:

```
coef.Pool.lm.cps3.1yt <- Pool.lm.cps3.1yt $qbar
sd.completecase.Pool.lm.cps3.1yt  <- apply(
  ODS[is.na(ODS$CPSS_t3)==0&is.na(ODS$CPSS_t1)==0&
        is.na(ODS$YouthTASC_t2)==0&is.na(ODS$TherTASC_t2)==0,
      c("CPSS_t3","CPSS_t1","YouthTASC_t2","TherTASC_t2")],
    2,sd)
coef.Pool.lm.cps3.1yt.standardized <- coef.Pool.lm.cps3.1yt[-1] *
  sd.completecase.Pool.lm.cps3.1yt[-1]/sd.completecase.Pool.lm.cps3.1yt[1]
coef.Pool.lm.cps3.1yt

coef.Pool.lm.cps3.1yt.standardized
```

#Model 2: DV = CAPS-CA, IV= Youth alliance T2, Therapist alliance T2, CAPS-CA T1

```
lm.caps.1yt <- lm.mids(CAPS_T3~ CAPS_T1+YouthTASC_t2 + TherTASC_t2,
              data=ImpODScaps.200)
Pool.lm.caps.1yt <- pool(lm.caps.1yt)
summary(Pool.lm.caps.1yt)
```

<u># Calculating standardized coefficients:</u>

```
coef.Pool.lm.caps.1yt <- Pool.lm.caps.1yt $qbar
sd.completecase. Pool.lm.caps.1yt <- apply(
  ODS[is.na(ODS$CAPS_T3)==0&is.na(ODS$CAPS_T1)==0&
      is.na(ODS$YouthTASC_t2)==0&is.na(ODS$TherTASC_t2)==0,
    c("CAPS_T3"," CAPS_T1","YouthTASC_t2","TherTASC_t2")],
  2,sd)
coef.Pool.lm.caps.1yt.standardized <- coef.Pool.lm.caps.1yt[-1] *
  sd.completecase. Pool.lm.caps.1yt[-1]/sd.completecase.Pool.lm.caps.1yt[1]
coef.Pool.lm.caps.1yt
sd.completecase.Pool.lm.caps.1yt
coef.Pool.lm.caps.1yt.standardized
```

#Model 3:
#DV = Child satisfaction, IV = Youth alliance T2, Therapist alliance T2, change CPSS #T1--T3

```
lm.chsat.CHcp1 <- lm.mids(Satisf.Child~ Ch_CPSSt1t3 + YouthTASC_t2 + TherTASC_t2,
              data=ImpODSchs.200)
Pool.lm.chsat.CHcp1 <- pool(lm.chsat.CHcp1)

summary(Pool.lm.chsat.CHcp1)
```

<u># Calculating standardized coefficients:</u>

```
coef.Pool.lm.chsat.CHcp1 <- Pool.lm.chsat.CHcp1 $qbar
sd.completecase.Pool.lm.chsat.CHcp1  <- apply(
  ODS[is.na(ODS$Satisf.Child)==0&

is.na(ODS$YouthTASC_t2)==0&is.na(ODS$TherTASC_t2)==0&is.na(ODS$Ch_CPSSt1t3)=
=0,
    c("Satisf.Child","YouthTASC_t2","TherTASC_t2", 'Ch_CPSSt1t3')],
  2,sd)
```

```
coef.Pool.lm.chsat.CHcp1.standardized <- coef.Pool.lm.chsat.CHcp1[-1] *
  sd.completecase.Pool.lm.chsat.CHcp1[-1]/sd.completecase.Pool.lm.chsat.CHcp1[1]
coef.Pool.lm.chsat.CHcp1
sd.completecase.Pool.lm.chsat.CHcp1
coef.Pool.lm.chsat.CHcp1.standardized
```

## Models including therapist – adolescent discrepancy:

# Model 4: DV = CPSS T3, IV = Therapist – adolescent discrepancy, CPSS T1

```
lm.cps3.1disc <- lm.mids(CPSS_t3~ CPSS_t1+TherMinYouth,
                data=ImpODScp3.200)
Pool.lm.cps3.1disc <- pool(lm.cps3.1disc)

summary(Pool.lm.cps3.1disc)

# Calculating standardized coefficients:
coef.Pool.lm.cps3.1disc <- Pool.lm.cps3.1disc $qbar
sd.completecase.Pool.lm.cps3.1disc  <- apply(
  ODS[is.na(ODS$CPSS_t3)==0&
      is.na(ODS$CPSS_t1)==0&is.na(ODS$TherMinYouth)==0,
    c("CPSS_t3","CPSS_t1","TherMinYouth")],
  2,sd)
coef.Pool.lm.cps3.1disc.standardized <- coef.Pool.lm.cps3.1disc[-1] *
  sd.completecase.Pool.lm.cps3.1disc[-1]/sd.completecase.Pool.lm.cps3.1disc[1]
coef.Pool.lm.cps3.1disc
sd.completecase.Pool.lm.cps3.1disc
coef.Pool.lm.cps3.1disc.standardized
```

# Model 5: DV = CAPS T3, IV = Therapist – adolescent discrepancy , CAPS T1

```
lm.caps.discr <- lm.mids(CAPS_T3~ CAPS_T1+TherMinYouth,
                 data=ImpODScaps.200)
Pool.lm.caps.discr <- pool(lm.caps.discr)
summary(Pool.lm.caps.discr)

# Calculating standardized coefficients:
coef.Pool.lm.caps.discr <- Pool.lm.caps.discr $qbar
sd.completecase.Pool.lm.caps.discr  <- apply(
  ODS[is.na(ODS$CAPS_T3)==0&
      is.na(ODS$CAPS_T1)==0&is.na(ODS$TherMinYouth)==0,
    c("CAPS_T3","CAPS_T1","TherMinYouth")],
  2,sd)
coef.Pool.lm.caps.discr.standardized <- coef.Pool.lm.caps.discr[-1] *
  sd.completecase.Pool.lm.caps.discr[-1]/sd.completecase.Pool.lm.caps.discr[1]
coef.Pool.lm.caps.discr
sd.completecase.Pool.lm.caps.discr
coef.Pool.lm.caps.discr.standardized
```

# Model 6: DV = Child satisfaction, IV = Therapist – adolescent discrepancy, #change CPSS T1-T3

```
lm.chsat.discCH <- lm.mids(Satisf.Child~ TherMinYouth+ Ch_CPSSt1t3,
                  data=ImpODSchs.200)
Pool.lm.chsat.discCH <- pool(lm.chsat.discCH)

summary(Pool.lm.chsat.discCH)

# Calculating standardized coefficients:
coef.Pool.lm.chsat.discCH <- Pool.lm.chsat.discCH $qbar
sd.completecase.Pool.lm.chsat.discCH  <- apply(

ODS[is.na(ODS$Satisf.Child)==0&is.na(ODS$TherMinYouth)==0&is.na(ODS$Ch_CPSSt1t
3)==0,
    c("Satisf.Child","TherMinYouth", 'Ch_CPSSt1t3')],
  2,sd)
coef.Pool.lm.chsat.discCH.standardized <- coef.Pool.lm.chsat.discCH[-1] *
  sd.completecase.Pool.lm.chsat.discCH[-1]/sd.completecase.Pool.lm.chsat.discCH[1]
coef.Pool.lm.chsat.discCH
sd.completecase.Pool.lm.chsat.discCH
coef.Pool.lm.chsat.discCH.standardized
```

#Comparing strength of correlations with bootstrap

```
# Correlations YouthTASC_t2 and TherTASC_t2 with
# Change CPSST1-T3, Change CAPST1-T3, and Child satisfaction:
cor(ODS[,c("YouthTASC_t2","Ch_CPSSt1t3","CAPS_ChT1T3","Satisf.Child")], use="p")
cor(ODS[,c("TherTASC_t2","Ch_CPSSt1t3","CAPS_ChT1T3","Satisf.Child")], use="p")

# New data set with only the analyzed variables included:
ODStab3ch <-
ODS[,c("YouthTASC_t2","TherTASC_t2","Ch_CPSSt1t3","CAPS_ChT1T3","Satisf.Child")]

#Bootstrap
cordiff <- function(frame1,dd) {
  frame2 <- frame1[dd,]
  cory <- cor(frame2[,c("YouthTASC_t2","Ch_CPSSt1t3"," CAPS_ChT1T3",
                  "Satisf.Child")], use="p")[2:4,1]
  cort <- cor(frame2[,c("TherTASC_t2","Ch_CPSSt1t3"," CAPS_ChT1T3",
                  "Satisf.Child")], use="p")[2:4,1]
  diff <- c(cory-cort)
  diff
} # end function cordiff

# sjekk med 100 replikasjoner:
nbootsjekk <- 100
boot1sjekk <- boot(data=ODStab3ch, statistic=cordiff, R=nbootsjekk)
boot1sjekk
```

```
nboot <- 10000
Sys.time()
boot1 <- boot(data=ODStab3ch, statistic=cordiff, R=nboot)
Sys.time()
boot1

# Confidence intervals:
boot1cicpss <- boot.ci(boot1, conf=c(.95,.99), type="bca", index=1)
boot1cicaps <- boot.ci(boot1, conf=c(.95,.99), type="bca", index=2)
boot1cisatis <- boot.ci(boot1, conf=c(.95,.99), type="bca", index=3)

# Summary:
boot1
boot1cicpss
boot1cicaps
boot1cisatis
```

<u>Note:</u>
'CPSS_t1' = PTSD symptoms, adolescent self-report, time 1
'CAPS_T1' = PTSD symptoms, clinical interview, time 1
'YouthTASC_t2' = adolesecent-rated alliance
'TherTASC_t2' = therapist-rated alliance
'TherMinYouth' = Therapist alliance score minus adolescent alliance score
'Satisf.Child' = Adolescent satisfaction with services
'Ch_CPSSt1t3' = Change in CPSS scores time 1 to time 3
CAPS_ChT1T3' = change in CAPS-CA time 1 to time 3
